# Enhancing Magnesium Bioactivity for Biomedical Applications: Effects of Laser Texturing and Sandblasting on Surface Properties

**DOI:** 10.3390/ma17204978

**Published:** 2024-10-11

**Authors:** Marjetka Conradi, Aleksandra Kocijan, Bojan Podgornik

**Affiliations:** Institute of Metals and Technology, Lepi pot 11, 1000 Ljubljana, Slovenia; aleksandra.kocijan@imt.si (A.K.); bojan.podgornik@imt.si (B.P.)

**Keywords:** biomaterial, microstructure, hardness, friction/wear, corrosion

## Abstract

Magnesium and its alloys, valued for their lightweight and durable characteristics, have garnered increasing attention for biomedical applications due to their exceptional biocompatibility and biodegradability. This work introduces a comparison of advanced and basic methods—laser texturing and sandblasting—on magnesium surfaces to enhance bioactivity for biomedical applications. Employing a comprehensive analysis spanning surface morphology, hardness, wettability, tribological performance, and corrosion behavior, this study elucidates the intricate relationship between varied surface treatments and magnesium’s performance. Findings reveal that both laser texturing and sandblasting induce grain refinement. Notably, sandblasting, particularly with a duration of 2 s, demonstrates superior wear resistance and reduced corrosion rates compared to untreated magnesium, thereby emerging as a promising approach for enhancing magnesium bioactivity in biomedical contexts. This investigation contributes to a deeper understanding of the nuanced interactions between diverse surface treatments and their implications for magnesium implants in chloride-rich environments, offering valuable insights for prospective biomedical applications.

## 1. Introduction

While magnesium and its alloys have long been recognized for their lightweight and durable properties in engineering applications, their potential extends beyond structural use [1,2,3]. Magnesium is also the fourth most common element in the human body, playing a key role in regulating biochemical reactions, supporting cell proliferation, and maintaining bone and mineral balance. Recent years have, therefore, seen a surge of interest in magnesium for its exceptional biocompatibility [4,5,6] and biodegradability [7,8,9]. These materials offer a unique combination of biocompatibility and mechanical properties, most similar to the bone in comparison to other metallic implants [7], making them increasingly relevant in the field of biomedical materials [2,10].

Ensuring stable bone fixation during the healing process and controllable degradation is paramount for implants, necessitating improved bioactivity of the implant surface to facilitate bonding between bone and implant [11]. In the past decades, researchers renewed their study of magnesium-based biodegradable implants thanks to advancements in processing technologies such as surface modification, thermomechanical processing, and alloying [12]. Various methods, including physico-chemical approaches, coatings, and surface texturing, have been proposed to enhance implant bioactivity [13,14,15,16,17,18,19]. These developments enable controlled corrosion rates for magnesium-based implants. Notably, surface roughness plays a pivotal role in cell adhesion and osteointegration [20,21], making surface texturing an appealing avenue for surface modification due to the possibility of improving bioactivity without chemically changing the surface of various materials for biomedical applications. Jahani B. et al. [22] modified the surface of the Ti13Nb13Zr alloy via grinding, polishing, machining, and sandblasting and studied the influence of roughness variation on mechanical properties, wettability, and cell attachment. Laser texturing of Ti-based alloys has also been accepted as a promising technique for surface modification via creating various surface textures (i.e., lines, crosshatch, dimples) that enable control of surface roughness and wettability and, under specific conditions, lead to improved cell attachment [23]. Laser texturing was also implemented for surface melting of Mg AZ31B alloy, leading to the chemical composition of the surface similar to the bone [24].

Despite the array of modern surface modification techniques available, sandblasting has garnered attention for its simplicity and cost-effectiveness in creating surface roughness with corundum particles (Al_2_O_3_), thereby facilitating osteointegration [25]. This technique allows for selective modification of surface properties, such as roughness, hardness, and wettability, through careful selection of particle size, shape, and kinetic energy, leading to plastic deformation of the subsurface layer.

The novelty of this work lies in comparing advanced and basic methods—laser texturing and sandblasting—on magnesium surfaces as potential approaches to enhance bioactivity through surface modification for biomedical applications. Following the idea of successful integration of the implant in the human body with the simplest approach possible, we focused on the correlation of its bioactivity with surface roughness, hardness, tribological, as well as corrosion properties. To fully understand the changes in the surface properties, we performed microstructural-crystallographic EBSD analysis and X-ray photoelectron spectroscopy (XPS) to evaluate the surface chemical composition. In summary, we provide a complete overview of the mechanical, tribological, and corrosion characteristics, coupled with surface chemical evaluation of magnesium. This enables a thorough assessment of the relationship between diverse surface treatments and their performance in chloride environments while also paving the way for implementing suitable surface treatments for biomedical applications. Overall, this research contributes to the advancement of implant technology and regenerative medicine by informing them of the selection of suitable surface treatments for biomedical applications.

## 2. Materials and Methods

### 2.1. Materials

The magnesium rod (Goodfellow, Peterlee, UK, 25 mm in diameter, 99.9% purity, as drawn, E = 42.5 GPa) was cut into discs of thickness 2 mm. The discs were further ground with SiC emery paper up to 4000 grit and finally diamond polished up to 1 µm. Prior to laser texturing and sandblasting, the polished samples were ultrasonically cleaned with ethanol and dried in warm air.

### 2.2. Surface Characterization

The surface morphology of as-received diamond-polished, laser-textured, and sandblasted magnesium samples was evaluated with scanning electron microscopy FIB-SEM ZEISS Crossbeam 550 SEM (Zeiss Group, Oberkochen, Germany) with an energy dispersive X-ray spectroscopy (EDS) analyzer.

A metallographic analysis was performed to evaluate the microscopic changes produced by laser treatment and sandblasting. For that purpose, the cross-sectioned samples were mounted in an epoxy resin, then ground, and finally polished using 1-µm diamond suspension and OPS (40-nm silica oxide colloidal suspension). The microstructural-crystallographic evaluation was conducted with a Hikari Super EBSD Camera.

Optical 3D metrology system, model Alicona Infinite Focus (Alicona Imaging GmbH, Bruker Alicona, Raaba, Austria), and IF-MeasureSuite (Version 5.1) software were used to analyze the average surface roughness, *Sa*, and *Rz* of samples. Four measurements on each sample were performed at magnification 20× with a lateral resolution of 0.9 μm and a vertical resolution of 50 nm. The size of the area analyzed was 0.5 × 0.5 mm^2^. To level the profile, corrections were made to exclude the general geometrical shape and possible measurement-induced misfits.

The X-ray photoelectron spectroscopy (XPS) analysis was performed using Versa Probe 3 AD (Stanford Nano Shared Facilities, Stanford, CA, USA) with a monochromatic Al Kα X-ray source. The analyzed area was spot with 200 μm diameter, and the analyzed depth was 3–5 nm. The survey spectra with three cycles were acquired at a pass energy of 224 eV, and a step of 0.5 eV, and high-resolution XPS spectra with at least 15 cycles were acquired at a pass energy of 69 eV and a step of 0.1 eV. During data processing, the carbon C 1 s peak with the binding energy (BE) of 284.7 eV, characteristic for C–C bonds, was used to correct possible charging effects. The accuracy of the binding energies was estimated to be ±0.2 eV. The measured spectra were processed with MultiPak 9.9.2 ULVAC-PHI software with Shirley background subtraction. Three different XPS measurements were performed on each sample, and the average composition was calculated. High-resolution spectra for C 1 s, O 1 s, and Mg 1 s were analyzed.

### 2.3. Laser Texturing

Surface texturing on diamond-polished magnesium substrate was performed with an LPKF nanosecond Nd-YAG laser with 1064 nm wavelength and an output power of 5 W. The system is equipped with a Scanlab SCANgine 14 processing head, which has an F theta-Ronar lens (F = 360 mm) and a double Galvano configuration. SAMLight SCAPS v3.5.5 software was used for programming specific textures, i.e., dimples. Based on our previous analysis, the pulse length was set to 0.5 ms, the pulse frequency 500 Hz, and the laser focus with a diameter of 30 µm was set on the Mg surface. The dimples with a diameter of 50 µm and depth of around 25 µm were arranged in a square formation with a center-to-center distance of 100 µm. Laser-texturing was performed in an argon atmosphere at room temperature without any post-treatment or post-polishing of the textured surface.

### 2.4. Sandblasting

**Figure 1:**
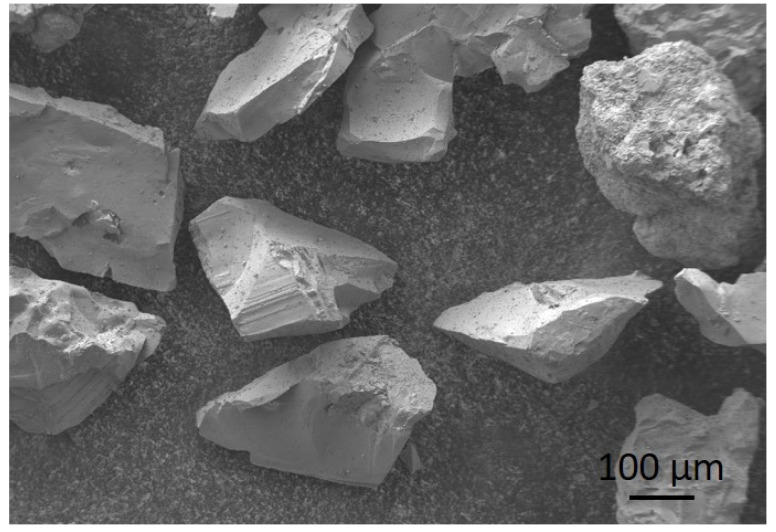
SEM image of Al_2_O_3_ particles used for sandblasting.

For surface modification via sandblasting, we used a sandblasting device (Gostol TST d.d., Tolmin, Slovenia), where the pressurized air in the device projected the abrasive Al_2_O_3_ particles (corundum, TESI Ltd., Bizeljsko, Slovenia) at a 45° angle onto the magnesium surface. The distance between the nozzle and the sample surface was 20 cm, and the air pressure was 6 bars. The sandblasting was carried out for 2 s, 5 s, 10 s and 30 s. The particle size varied between 212–250 µm, and the shape of the particles is shown in Figure 1.

### 2.5. Wettability Measurements

The water-contact angles at room temperature and ambient humidity were measured and the results were analyzed with a surface-energy-evaluation system (Advex Instruments s.r.o., Brno, Chech Republic). Four measurements with 5 μL water droplets were performed on different spots of each surface to minimize the influence of roughness and gravity.

### 2.6. Hardness

The nanoindentation measurements were conducted using the Hysitron TS 77 instrument from Bruker (Billerica, MA, USA), employing a diamond Berkovich probe throughout all experiments. A standard quasi-static load function was applied, featuring 5 s of loading, a 2-s holding period, and 5 s of unloading. The maximum load applied was 8000 μN. Experimental tests were systematically performed in a grid pattern with a 5 μm spacing. Approximately 60 measurements were acquired for each sample at various distances from the sample surface within a sample cross-section. The nanoindentation hardness (H) and elastic modulus (E) were subsequently computed from the load–displacement curve, employing the Oliver and Pharr model [26].

### 2.7. Potentiodynamic Measurements

Potentiodynamic measurements for polished (DP), laser-textured (LT), and sandblasted magnesium samples (SB 2 s, SB 5 s, SB 10 s, and SB 30 s) were performed in simulated physiological Hank’s solution at room temperature and pH = 7.8. Hank’s solution contained 8 g/L NaCl, 0.40 g/L KCl, 0.35 g/L NaHCO_3_, 0.25 g/L NaH_2_PO_4_ × 2H_2_O, 0.06 g/L Na_2_HPO_4_ × 2H_2_O, 0.19 g/L CaCl_2_ × 2H_2_O, 0.41 g/L MgCl_2_ × 6H_2_O, 0.06 g/L MgSO_4_ × 7H_2_O and 1 g/L glucose (all Merck chemicals). The potentiodynamic curves were obtained by using the BioLogic SP-300 Model instrument (BioLogic, Seyssinet-Pariset, France) and EC-Lab V11.27 software. The three-electrode electrochemical system was used with the test specimen as a working electrode, a saturated calomel electrode as a reference electrode, and a platinum mesh as a counter electrode. The samples were stabilized for an hour at the open-circuit potential. The scan rate was 1 mV/s. To obtain statistically relevant results, all the measurements were repeated 3 times.

### 2.8. Tribological Testing

Tribological properties were evaluated with the help of a TRIBOtechnic friction testing tribometer in a ball-on-flat contact configuration under a reciprocating sliding motion. Reciprocating sliding, although not providing uniform and constant contact conditions, was selected to more closely simulate the conditions typical of biomedical applications [21]. Tests on the diamond-polished, laser-textured, and sandblasted magnesium discs were performed under dry and fully flooded lubricated conditions in simulated physiological Hank’s solution at ambient conditions (RH = 40%, T = 22 °C). Each test was repeated at least three times. The total sliding distance of 1 m was achieved under 5 N normal load (contact pressure of 400 MPa) and at an average sliding speed of 5 mm/s. An inert ceramic alumina ball (Al_2_O_3_, E = 380 GPa) with a diameter of 10 mm was used as a stationary counter-body and loaded against the magnesium disc. The load and ball diameter were selected based on the contact conditions experienced in metal joint replacements. Typical contact pressures for metal hip and knee joint replacements range between 50 and 100 MPa [27]. However, misalignments, defects, and damages to the contact surfaces can easily lead to 3–4 times higher contact pressures. In order to simulate extreme and most critical contact conditions, a contact pressure of 400 MPa was selected for this study. Furthermore, the wear volumes were measured by the three-dimensional (3D) Focus-Variation measuring tool Alicona InfiniteFocus G4 (Bruker Alicona, Raaba, Austria).

## 3. Results and Discussion

### 3.1. Surface Morphology

**Figure 2:**
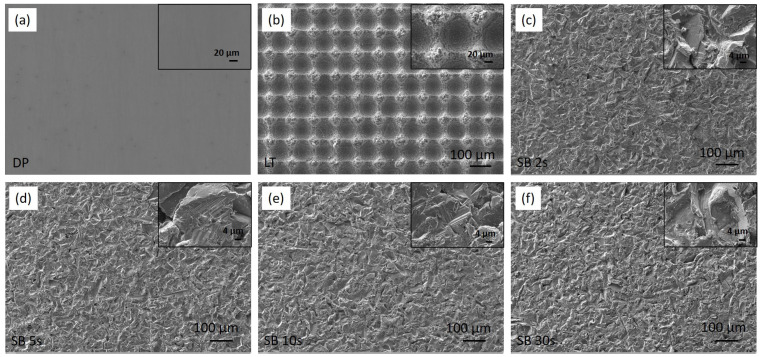
SEM images of magnesium surfaces under investigation: diamond-polished (**a**), laser-textured (**b**), sandblasted for 2 s (**c**), sandblasted for 5 s (**d**), sandblasted for 10 s (**e**), and sandblasted for 30 s (**f**). The inset images show the details of the surfaces’ morphology at higher magnification.

**Figure 3:**
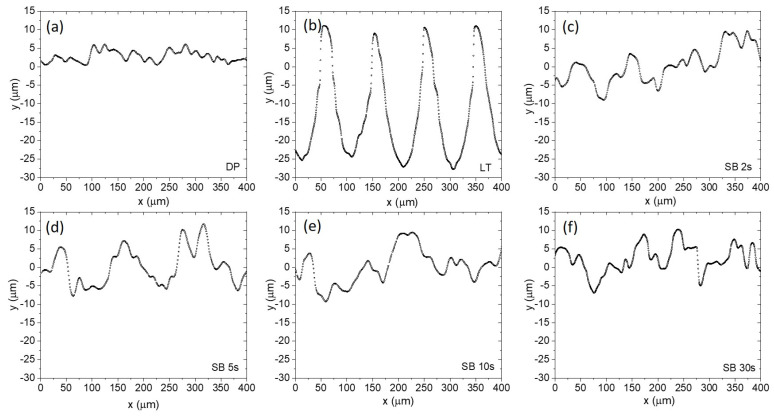
Surface profiles of diamond-polished (**a**), laser-textured (**b**), sandblasted for 2 s (**c**), sandblasted for 5 s (**d**), sandblasted for 10 s (**e**), and sandblasted for 30 s (**f**).

Figure 2 shows the SEM images, and Figure 3 shows the corresponding surface profiles of the Mg surfaces under investigation: diamond polished (DP) before surface treatment (a), laser-textured (LT) (b), and sandblasted (SB) surfaces (c–f). The unmodified surface is diamond polished up to 1 µm; therefore, no visible grinding marks are observed. For laser-textured surface, the morphology was defined with specific laser parameters (power, frequency, speed, repetitions), resulting in a square-like configuration of dimples with a depth of approximately 25 µm, a diameter of 50 µm, a center-to-center distance of 100 µm and about 10 µm ablated material around dimples’ perimeter (Figure 3b). Sandblasted surfaces show a significant change in surface morphology with increased roughness due to the plastic deformation being correlated with the impact of corundum particles. The degree of plastic deformation is associated with the processing parameters as described in the Experimental section. Here, we varied the time of sandblasting from 2 s, 5 s, 10 s, and up to 30 s, which led to an increase in the average surface roughness, *Sa,* as well as *Sz* with increasing time. As shown in Table 1, LT and SB surfaces have significantly higher Sa and *Sz* in comparison to the DP sample. This is also reflected in the surface profile analysis (Figure 3) and is responsible for specific tribological properties of LT and SB surfaces, as described in Section 3.5.

**Figure 4:**
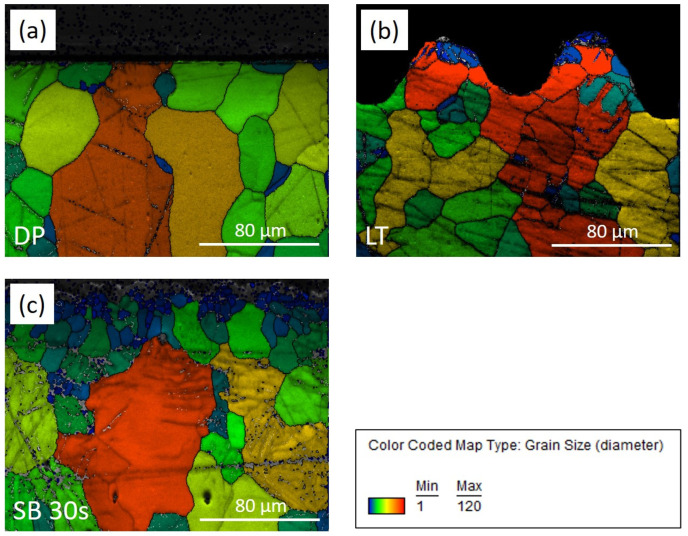
EBSD band contrast image overlapping with the EBSD phase map for diamond-polished (**a**), laser-textured (**b**), and sandblasted 30 s (**c**) magnesium surface.

**Figure 5:**
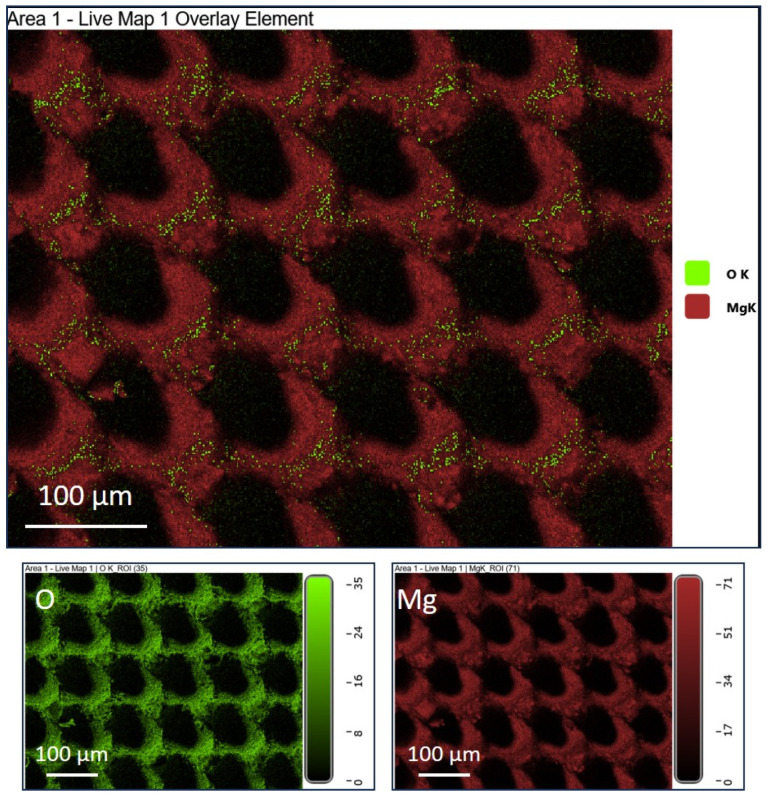
EDS mapping of laser textured surface indicating increased surface oxidation of the ablated area.

To understand the influence of the laser texturing and sandblasting on microstructural-crystallographic characteristics, we performed EBSD mapping of the cross-section of the laser-textured (LT) and sandblasted surface, SB 30 s in comparison to unprocessed diamond-polished (DP) magnesium surface (Figure 4). As reported by the manufacturer, the magnesium rod was produced as-drawn and characterized with a typical magnesium microstructure consisting of non-uniform grains ranging from 10 µm to a few tenths of micrometers (Figure 4a). Laser texturing with the selected parameters affected a very thin layer where grain refinement was observed due to the melted and rapidly solidified material (Figure 4b). Grain refinement is concentrated at the peaks of the dimples in about 10 µm thick surface layer where also the appearance of several twins is observed. Grain refinement is also observed at the bottom of the dimples, however, to a lesser extent, in a thin ~1 µm layer. Laser textured surface also shows increased surface oxidation of the ablated area, as revealed by EDS mapping (Figure 5). In the case of a sandblasted surface, high kinetic energy during sandblasting induces plastic deformation, leading to grain refinement in the subsurface layer extending down to 30 µm and the formation of twins (Figure 4c). This intensifies with the sandblasting time.

### 3.2. Surface Hardness

**Figure 6:**
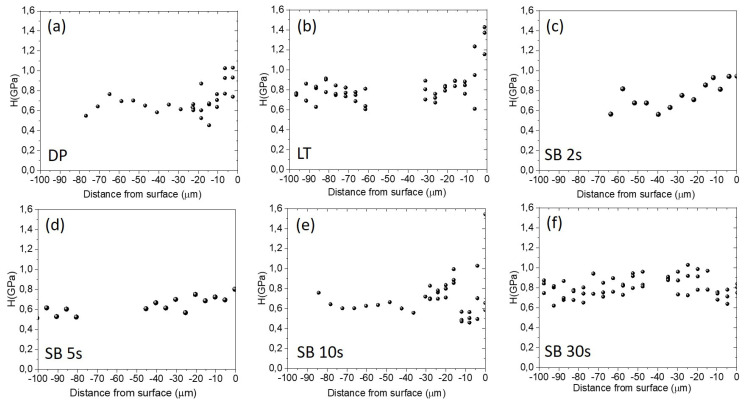
Hardness (GPa) variation with the distance from the sample’s surface into the bulk for diamond-polished (**a**), laser-textured (**b**), and sandblasted surfaces from 2 s–30 s (**c**–**f**).

The variation of surface hardness as a function of distance from the sample’s surface into the bulk is presented in Figure 6. The results indicate that the highest hardness of around 1.5 GPa, extending 10–20 µm into the surface, was measured for the laser-textured sample, followed by the samples sandblasted for 10 s and 30 s. Other sandblasted samples, SB 2 s and SB 5 s, show comparable hardness to our reference diamond-polished surface, around 1 GPa. In all surface-treated samples, we, however, observe a slight decrease in hardness when we move away from the surface. This indicates the surface hardening effect in a thin surface layer of around 10–30 µm due to either laser-texturing or sandblasting, with longer sandblasting times resulting in higher intensity. This is in correlation to the microstructural analysis of the samples (Section 3.1). It has been shown that the surface hardness of magnesium is increased mostly due to grain refinement after surface modification [28,29,30]. Laser texturing modifies the microstructure of Mg due to rapid heating and cooling effects, resulting in recrystallization, as well as local surface oxidation. The plastic deformation induced by sandblasting also leads to recrystallization, resulting in reduced grain size in the subsurface layer.

A comparison of the Young modulus, E, of the surface treated samples to the reference diamond-polished magnesium reveals increased stiffness and resistance to deformation for all treated samples. The highest E, around (60 ± 4) GPa, is observed for the SB 10 s sample, followed by the SB 30 s sample with E = (51 ± 4) GPa. On the other hand, LT, SB 2 s, and SB 5 s surfaces have a Young modulus of the same order, around 45 GPa; however, still higher than the Young modulus measured on the untreated DP surface, E = (38 ± 2) GPa.

### 3.3. XPS

**Figure 7:**
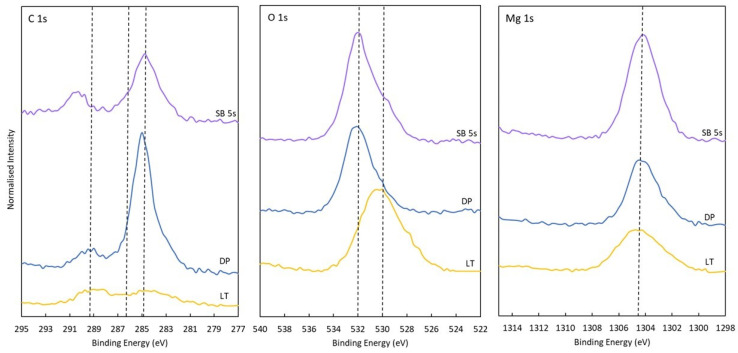
XPS spectra C 1 s, O 1 s, and Mg 1 s from polished (DP), laser-textured (LT), and sandblasted for 5 s (SB 5 s) magnesium samples.

The surface composition and the oxidation state of the elements in the oxide layer on DP, LT, and SB 5 s magnesium samples were determined by using XPS analysis. Mainly, three elements were present on the surface: carbon, oxygen and magnesium. The chemical composition derived from XPS analyses, as well as C/O and O/Mg ratios, are presented in Table 2. High-resolution spectra for C 1 s, O 1 s, and Mg 1 s are shown in Figure 7. A considerable change in chemical composition was observed for LT and SB 5 s samples. The increase in oxygen and magnesium content is evident for both LT and SB 5 s treatments.

The high-resolution spectrum for C 1 s revealed that carbon mainly originated from three components. The first component at 284.7 eV was related to C–C/C–H bonds. A second component at 286.5 eV was attributed to C–OH bonds, and the third component at 289.2 eV corresponded to the O=C–O/CO_3_ component. The O 1 s spectrum showed the main peak on the DP sample at 532.0 eV, corresponding to C–O, CO_3_, and OH bonds. After the surface treatments, a shift to 530.0 eV was observed, which is characteristic of the formation of MgO. This is in correlation with a lower carbon content and a lower O/Mg ratio for surface-treated samples.

### 3.4. Wettability

We compared the wettability of the diamond-polished, laser-textured, and sandblasted surface. We completed four measurements of contact angles, θ, in Hank’s solution on each surface and used them to calculate the average contact angle values listed in Table 3. We can see that our reference, a diamond-polished surface, is moderately hydrophilic with CA 75°. Laser-textured surface is superhydrophobic with CA above 150°. The wettability of sandblasted surfaces is related to the time of sandblasting. The surface of SB 2 s is neutrally wet with a contact angle slightly above 90°. With the increasing time of sandblasting, the contact angle decreases, and the surfaces become hydrophilic, which is also correlated to increased average surface roughness (Table 1). This suggests that the sandblasted surfaces are in the Wenzel wetting regime, allowing a controllable surface wettability according to the chosen sandblasting parameters [31].

As the contact angles were only available for water, an equation-of-state approach [32,33] was used to calculate the surface energies with Equation (1):

(1) $$COS\left(\theta \right)=-1+2\sqrt{\frac{\gamma s}{\gamma l}e^{-\beta \left(\gamma l-\gamma s\right)2}}$$

For a given value of the surface tension of the probe liquid *γ*_l_ (i.e., for water *γ_l_* = 72.8 mN/m [34]) and *θ^W^* measured on the same solid surface, the constant *β* and the solid surface tension *γ_s_* values were determined using the least-squares analysis technique. For the fitting with Equation (1), a literature value of *β* = 0.0001234 (mJ/m^2^)^−2^ was used, as weighted for a variety of solid surfaces [35].

We should also stress that magnesium surfaces in water exhibit Lewis acid-base behavior, with magnesium acting as a Lewis acid by accepting electron pairs from water, which acts as a Lewis base. This interaction results in the formation of magnesium hydroxide and hydrogen gas, highlighting the acid-base dynamics at the magnesium–water interface [36].

### 3.5. Tribological Evaluation

#### 3.5.1. Friction

**Figure 8:**
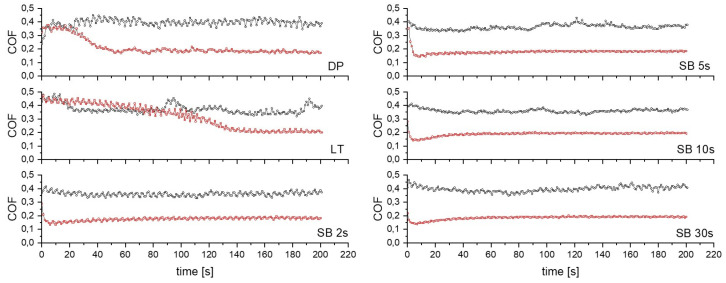
Comparison of friction curves for untreated (DP), laser-textured (LT), and sandblasted (SB) surfaces measured in air (black ○) and under lubrication with Hank’s solution (red □).

**Figure 9:**
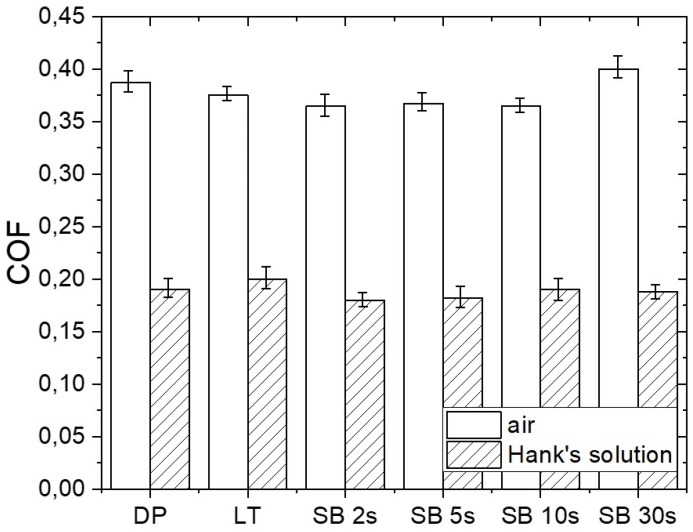
Comparison of steady-state coefficient of friction (COF) for untreated (DP), laser-textured (LT), and sandblasted (SB) magnesium surfaces measured in air and under lubrication with Hank’s solution.

Figure 8 presents typical friction curves in air and under lubrication with Hank’s solution for diamond-polished Mg before surface treatment (DP), laser-textured (LT), and sandblasted (SB) Mg surfaces. For sliding in air, the DP surface shows relatively stable friction with a typical running-in shape for metals with a thin adsorbed layer of contamination on the contact surface. The steady-state COF for DP is around 0.4 (Figure 9). On the contrary, all surface-modified Mg surfaces indicate a certain running-in stage related to surface topography wear and contact surface accommodation before achieving a steady-state COF. Friction on the LT surface in air starts with a high COF plateau (~0.45) due to rough surface and reduced contact area, followed by a rather abrupt decrease into the steady state with COF around 0.35. Initial friction of sandblasted surfaces starts at about 0.4, and through asperities, wear and smoothening slowly decrease toward a steady-state COF of 0.35–0.40. The duration of the running-in stage correlates well with the time of sandblasting. SB 2 s, SB 5 s, and SB 10 s surfaces result in approximately the same steady-state COF of around 0.35, while SB 30 s sample results in COF around 0.40 due to more pronounced microstructure refinement and deeper plastic deformation and surface hardening effect obtained at 30 s sandblasting time.

In Hank’s solution, a distinctive running-in period is observed for all Mg surfaces; however, it differs for DP and LT samples as compared to SB samples. Friction of DP and LT in Hank’s solution starts with a high COF plateau of around 0.35 and 0.45, respectively. COF then slowly decreases to a steady-state value of about 0.20. This indicates a transition from boundary into mixed lubrication for both DP and LT. However, we should point out that running-in and the transition into a mixed lubrication regime is much wider for LT surface, which can be related to specific topography with the highest surface roughness and hardness, laser-induced formation of surface oxides, and the superhydrophobic nature of the laser modified surface making it more difficult to smoothen-out and/or form lubrication film. Friction for the sandblasted surfaces, on the contrary, shows an immediate drop from a high initial value of ~0.3 down to the steady-state value of ~0.18. However, longer SB time results in lower initial friction due to more resistant surface.

#### 3.5.2. Wear

**Figure 10:**
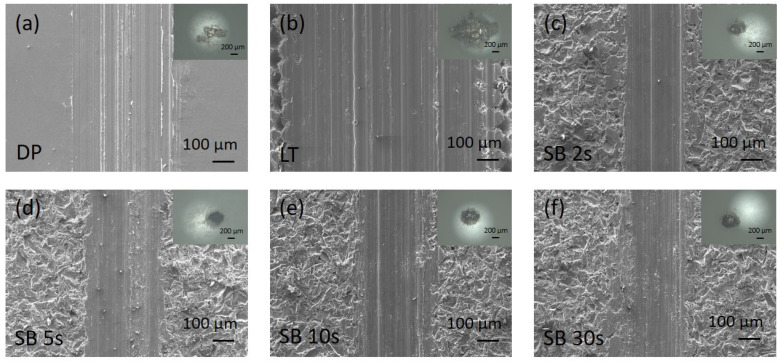
Wear scar SEM micrographs in air for untreated (**a**), laser-textured (**b**), and sandblasted (**c**–**f**) magnesium surfaces. The insets show the wear scar on the counter body, Al_2_O_3_ ball.

**Figure 11:**
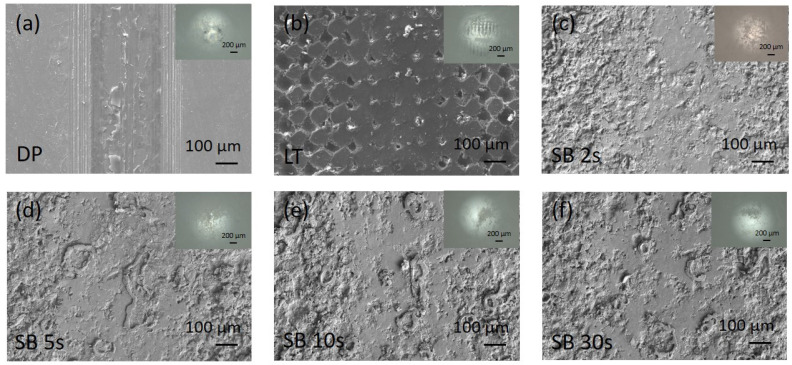
SEM micrographs of wear scars under lubrication in Hank’s solution for untreated (**a**), laser-textured (**b**), and sandblasted (**c**–**f**) magnesium surfaces. The insets show the wear scar on the counter body, Al_2_O_3_ ball.

In terms of wear, the prevailing wear mechanism for all magnesium surfaces was sliding abrasive wear and adhesion of worn Mg material on the Al_2_O_3_ counter-ball surface, as depicted in Figure 10 and Figure 11. Wear scars in air are very well defined, with typical abrasive scratches and plowing, sharp edges (Figure 10), and characteristic semi-elliptical depth profile for all the samples. In Hank’s solution, the wear, scars, and damage are much shallower and smaller. Especially on sandblasted samples, wear scars show a typical surface smoothening profile and are less distinctive, mostly due to the degradation of magnesium in a chlorine solution (Figure 11). The insets in Figure 10 and Figure 11 show wear scars with adhered Mg wear debris on the counter-body.

**Figure 12:**
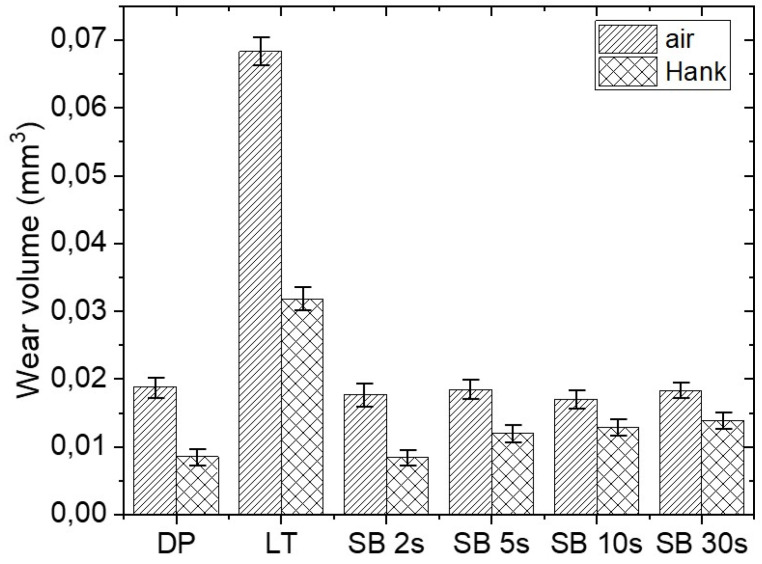
Wear volumes of untreated, laser-textured, and sandblasted magnesium surfaces in air and under lubrication in Hank’s solution.

Wear volumes for magnesium samples were measured by the 3D Focus-Variation measuring tool Alicona InfiniteFocus G4, defined as a loss of material below the surface reference plane and are presented in Figure 12. The lowest and comparable wear volumes were observed for sandblasted surfaces, slightly lower in comparison to untreated Mg surfaces, which is provided through plastic deformation and surface hardening. However, longer sandblasting times lead to increased surface roughness, longer running-in, and a higher risk of forming additional wear particles, as well as the potential presence of trapped sandblasting particles. Thus, the lowest wear volume was measured for SB 2 s surface, for both unlubricated (air) and Hank’s solution lubricated reciprocating sliding contact, ~0.017 mm^3^ in air and ~0.008 mm^3^ in Hank’s solution.

The highest wear volume was, on the other hand, found for the laser-textured sample: ~0.068 mm^3^ in air and ~0.032 mm^3^ in Hank’s solution. Laser-texturing intensified wear of the magnesium surface in comparison to the untreated DP surface, mostly due to the formation of hardened rough ablated surface and the presence of oxides, which lead to reduced contact area, increased contact stresses within the contact [37], and transfer from two-body to three-body abrasive wear [38].

### 3.6. Electrochemical Evaluation

**Figure 13:**
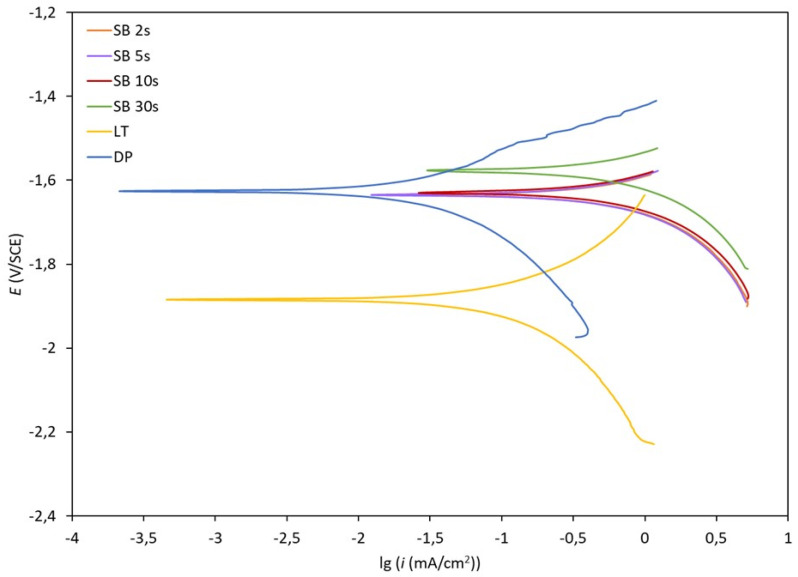
Potentiodynamic curves for polished (DP), laser-textured (LT), and sandblasted for 2–30 s (SB 2 s, SB 5 s, SB 10 s, and SB 30 s) magnesium samples measured in simulated physiological Hank’s solution at pH = 7.8 and room temperature.

The corrosion behavior of DP, LT, SB 2 s, SB 5 s, SB 10 s, and SB 30 s magnesium samples in simulated physiological solution was evaluated by using potentiodynamic polarization curves (Figure 13). Corresponding electrochemical parameters, i.e., corrosion potential (Ecorr), corrosion current density (icorr), and corrosion rate (vcorr), are shown in Table 4. The vcorr and the icorr values were calculated according to the ASTM G102-89 standard (2015) [39]. The diamond-polished sample exhibited the lowest icorr and vcorr values; slightly higher values were observed for the laser-textured sample. A significant increase of icorr and vcorr values was observed for all sandblasted samples compared to DP and LT.

As mentioned above, surface modification enhances the surface roughness as well as the surface hardness. Analysis of the LT surface showed a recrystallization effect due to melting and rapid solidification of magnesium. In the case of SB samples, the high kinetic energy induced by sandblasting produces plastic deformation in the subsurface layer of the treated magnesium and also induces the formation of voids and microcracks. The recrystallization induced by surface treatments generates a reduction of grain size and formation of twins, which, on the one hand, leads to increased surface hardness and, on the other hand, has a negative effect on corrosion resistance [40]. The XPS results showed that the highest O/Mg ratio was observed on the DP sample, which also exhibited the highest corrosion resistance. The decreased corrosion resistance of LT and SB samples can also be ascribed to the decreased O/Mg ratio on the surface. However, the increase in corrosion rate is less pronounced for the LT sample, most probably due to the superhydrophobic nature and high surface area (10 times higher roughness) of the LT surface. This is in agreement with literature data reporting that laser surface modification techniques improve mechanical properties and cell adhesion but can have a negative effect on corrosion behavior [12].

## 4. Conclusions

This study offers a comprehensive understanding of the intricate relationship between diverse surface treatments, i.e., sophisticated LT and basic sandblasting methods, and their implications for magnesium implants. Key insights include the importance of considering mechanical, tribological, and corrosion characteristics in chloride environments when selecting appropriate surface treatments.

SEM and EBSD analyses have revealed that both laser texturing and sandblasting significantly modified the surface morphology as well as microstructure through grain refinement. These surface modifications led to an increase in surface hardness in comparison to untreated Mg, which was most pronounced for the LT sample. In terms of wettability, LT surfaces exhibited superhydrophobic behavior, while sandblasted surfaces demonstrated controllable hydrophilic wettability based on processing parameters. Tribological evaluations highlighted the influence of surface treatments on friction and wear behavior, with laser surface texturing without additional surface polishing resulting in increased wear, while short low-intensity sandblasting showed superior friction and wear resistance.

Corrosion resistance was affected by surface modifications, with LT and SB surfaces showing increased corrosion rates compared to the untreated diamond-polished surface. The superhydrophobic nature of LT surface and specific sandblasting parameters influenced corrosion rates differently, offering insights into tailored surface treatments.

Overall, a basic sandblasting method, particularly for 2 s, emerged as a promising technique, exhibiting superior wear resistance, controllable wettability, and relatively lower corrosion rates, making it well-suited for biomedical applications. These findings provide a foundation for further research on optimizing simple rather than advanced surface modification techniques for magnesium implants in biomedical applications. Future studies could explore the long-term in vivo performance and biocompatibility of optimized surfaces to validate their potential clinical use.

## Figures and Tables

**Table 1:** Average surface roughness, *Sa* (µm) and *Sz* (µm) for untreated (DP) laser-textured (LT) and sandblasted (SB) magnesium surfaces.

Sample	*Sa* (µm)	*Sz* (µm)
DP	0.25 ± 0.02	1.77 ± 0.02
LT	17.3 ± 1.2	69.1 ± 0.9
SB 2 s	1.52 ± 0.14	9.53 ± 0.07
SB 5 s	1.61 ± 0.15	11.71 ± 0.14
SB 10 s	1.71 ± 0.16	11.48 ± 0.15
SB 30 s	1.73 ± 0.16	12.71 ± 0.14

**Table 2:** Chemical composition in at% of surfaces derived from XPS analyses.

Sample	Chemical Composition (at%)				
	C	O	Mg	C/O	O/Mg
DP	53.50	42.81	3.69	1.25	11.62
SB 5 s	32.46	61.14	6.40	0.53	9.56
LT	36.55	56.98	6.47	0.64	8.81

**Table 3:** Static water contact angles (θ) for untreated (DP), laser-textured (LT), and sandblasted (SB) magnesium surfaces. Static contact angles were measured with water, and the corresponding surface energies were calculated using an equation-of-state approach.

Sample	*Ɵ* (°)	*γs* [mN/m]
DP	75 ± 2	36.6 ± 0.2
LT	>150 (superhydrophobic)	1.1 ± 0.1
SB 2 s	94 ± 3	26.7 ± 0.2
SB 5 s	77 ± 2	37.6 ± 0.2
SB 10 s	68 ± 2	43.0 ± 0.2
SB 30 s	60 ± 2	47.6 ± 0.2

**Table 4:** Electrochemical parameters calculated from the potentiodynamic curves.

Sample	*E*_corr_ (V vs. SCE)	*i*_corr_ (µA/cm^2^)	*v*_corr_ (mm/year)
DP	−1.64 ± 0.03	35.4 ± 0.2	1.62 ± 0.01
LT	−1.89 ± 0.04	63.7 ± 0.3	2.91 ± 0.01
SB 2 s	−1.62 ± 0.03	334.7 ± 0.5	15.29 ± 0.03
SB 5 s	−1.62 ± 0.03	293.8 ± 0.5	13.43 ± 0.03
SB 10 s	−1.61 ± 0.03	382.5 ± 0.5	17.48 ± 0.04
SB 30 s	−1.56 ± 0.02	373.5 ± 0.5	17.07 ± 0.04

## Data Availability

Data are available in the open repository DiRROS with PID http://hdl.handle.net/20.500.12556/DiRROS-18441, accessed on 11 September 2024.
